# Valvulo-Arterial Impedance in Patients with Severe Aortic Stenosis and Bicuspid Aortic Valve

**DOI:** 10.3390/jcdd13040163

**Published:** 2026-04-09

**Authors:** Chun Kit Ng, Pilar Lopez Santi, Marie-Ange Fleury, Jingjing He, Nadeem Elmasry, Steele C. Butcher, Marie-Annick Clavel, Philippe Pibarot, Jeroen J. Bax, Nina Ajmone Marsan

**Affiliations:** 1 Department of Cardiology, Leiden University Medical Center, 2300RC Leiden, The Netherlands; c.k.ng@lumc.nl (C.K.N.); m.p.lopez_santi@lumc.nl (P.L.S.); j.he@lumc.nl (J.H.); n.m.h.e.elmasry@lumc.nl (N.E.); s.c.butcher@lumc.nl (S.C.B.); j.j.bax@lumc.nl (J.J.B.); 2 Cardiology, Institut Universitaire de Cardiologie et de Pneumologie de Québec, Université Laval, G1V 4G5 Québec, QC, Canada; marie-ange.fleury.1@ulaval.ca (M.-A.F.); marie-annick.clavel@criucpq.ulaval.ca (M.-A.C.); philippe.pibarot@med.ulaval.ca (P.P.); 3 Department of Cardiology, Turku Heart Center, University of Turku and Turku University Hospital, 20520 Turku, Finland

**Keywords:** aortic stenosis, bicuspid aortic valve, valvulo-arterial impedance

## Abstract

Background: Risk stratification in patients with bicuspid aortic valve (BAV) and severe aortic stenosis (AS) remains challenging. Valvulo-arterial impedance (Zva), an integrated marker of global left ventricular (LV) afterload, has shown prognostic value in tricuspid AS; however, data in BAV are limited. This study aimed to evaluate the association of Zva with LV remodeling, symptoms, and all-cause death in patients with BAV and severe AS. Methods: In this retrospective, two-center cohort study, 147 patients with severe AS and BAV were included. Zva was calculated at the time of the first echocardiographic diagnosis of severe AS. The study endpoint was all-cause mortality. Results: Over a median follow-up of 9.8 years, 24 patients (16%) died. A Zva threshold of 5 mmHg/mL/m^2^ was identified as optimal by ROC analysis. Patients with Zva ≥ 5 mmHg/mL/m^2^ showed higher mortality rates (29% vs. 10%; *p* = 0.003), more advanced symptoms (NYHA III-IV: 41% vs. 9%; *p* < 0.001), adverse LV remodeling, lower LVEF (60% (IQR 36–66) vs. 66% (IQR 61–71); *p* = 0.001), and worse LV global longitudinal strain (14.8% ± 2.7 vs. 16.5% ± 3.0; *p* = 0.016). Zva ≥ 5 mmHg/mL/m^2^ was independently associated with worse long-term survival after adjustment (HR 2.885; 95% CI 1.119–7.438; *p* = 0.028). Conclusions: Among patients with BAV and severe AS, an increased Zva was associated with more advanced symptoms, adverse LV remodeling, impaired LV systolic function, and worse long-term survival, and might therefore help in risk stratification of these patients.

## 1. Introduction

Bicuspid aortic valve (BAV) is the most common congenital cardiac valve anomaly, with an estimated prevalence of 0.5% to 2% in the general population. It is associated with an earlier onset of degenerative aortic valve disease, including aortic stenosis (AS), aortic regurgitation, or both [1,2]. Despite advances in imaging and therapeutic interventions, risk stratification in BAV-related severe AS remains challenging.

Valvulo-arterial impedance (Zva) has been proposed as a novel parameter to improve risk stratification in tricuspid aortic valve (TAV) related AS by accounting for the combined valvular and arterial hemodynamic burden imposed as afterload on the left ventricle (LV). Zva is measured by relating systolic arterial blood pressure (SBP) and aortic valvular mean pressure gradient (AV MPG) to indexed stroke volume (SVi). In patients with TAV-related AS, increased Zva has been associated with more advanced LV remodeling, worse LV function, and more symptoms, even after valve intervention [3]. Importantly, increased Zva is also associated with increased mortality among asymptomatic patients with preserved LV ejection fraction (LVEF) [3,4,5].

However, evidence regarding the applicability of Zva in patients with BAV-related AS is currently limited. Distinct from TAV, BAV commonly presents as a valvulo-aortopathy with asymmetric valve morphology, eccentric flow jets, and altered arterial properties [6]. Furthermore, patients with BAV tend to be younger and have fewer cardiovascular comorbidities, such as arterial hypertension, which may influence both Zva values and their prognostic implications compared with TAV cohorts [7]. Therefore, the aim of this study was to assess (i) the optimal Zva threshold associated with all-cause death and (ii) the association of Zva with symptoms, LV remodeling, and LV systolic function in patients with BAV and severe AS.

## 2. Materials and Methods

### 2.1. Patient Population and Data Collection

This retrospective, observational cohort study included patients who presented with a first echocardiographic diagnosis of BAV and severe AS at two tertiary centers: Leiden University Medical Center (Leiden, The Netherlands) and Québec Heart and Lung Institute (Québec, QC, Canada).

Severe AS was defined as an aortic valve area (AVA) less than 1.0 cm^2^ or an indexed AVA less than 0.6 cm^2^/m^2^ [8,9]. Zva was calculated as (SBP + AV MPG)/Svi [4]. The echocardiographic measurements were performed at the first available transthoracic echocardiogram, diagnosing severe AS. Patients with incomplete data for the calculation of the Zva, previous aortic valve replacement (AVR), or significant aortic regurgitation were excluded from the study. Also, patients in whom SBP measurements were not available in temporal proximity to the baseline echocardiographic assessment were excluded from the study. Medical history, baseline demographics, and clinical data of each patient were collected from the electronic medical records. The study was approved by the institutional review board at each center, and, due to its retrospective nature, the need for written consent was waived.

### 2.2. Echocardiography

Transthoracic echocardiography (TTE) was performed using commercially available equipment (Vivid 7, E9, and E95 systems, GE-Vingmed, Horten, Norway; EPIQ, Philips, Mississauga, ON, Canada) in the left lateral decubitus position. Images were digitally stored for offline analysis with EchoPAC (V7.0 software from GE Vingmed Ultrasound, Horten, Norway) or Xcelera (Philips, Mississauga, ON, Canada) and retrospectively analyzed by an experienced physician at each institution. The first TTE confirming the presence of severe AS was considered the baseline. BAV morphology was classified according to the system proposed by Sievers and Schmidtke [10].

From the apical three- or five-chamber views, continuous-wave Doppler recordings were obtained to estimate peak aortic jet velocity. AV MPG was calculated using the Bernoulli equation [11]. AVA was calculated with the continuity equation using velocity-time integrals (VTI) of the left ventricular outflow tract (LVOT) and AV, and indexed for body surface area (BSA) calculated by the formula of Du Bois [12]. LV SV was derived from the LVOT diameter, measured in parasternal long-axis view at mid-systole, and the LVOT VTI, and was subsequently indexed for BSA [8].

In the parasternal long-axis view, LV linear dimensions were assessed, and LV mass (LVM) was calculated using Devereux’s formula and indexed for BSA (LVMi) [12,13]. Normal LVMi was defined as LVMi ≤ 95 g/m^2^ for women and LVMi ≤ 115 g/m^2^ for men [12]. Relative wall thickness (RWT) was calculated with the formula: (2 × posterior wall thickness)/(LV end-diastolic diameter), and a cut-off of >0.42 was used to define concentric LV remodeling [12]. LVMi and RWT were subsequently used to categorize patients into four LV remodeling patterns: (1) normal geometry: normal LVM and RWT ≤ 0.42; (2) concentric remodeling: normal LVM and RWT > 0.42; (3) concentric hypertrophy: increased LVM and RWT > 0.42; and (4) eccentric hypertrophy: increased LVM and RWT ≤ 0.42 [12]. LV end-diastolic and LV end-systolic volumes were measured from the apical two- and four-chamber views, and LVEF was calculated using Simpson’s biplane method [12]. LV strain was measured by automatic tracing of the LV endocardial border using images from the apical two-, three-, and four-chamber views, with manual corrections if necessary. The LV global longitudinal strain (GLS) was calculated by averaging all segmental peak strain values and was expressed as absolute values [12]. All remaining measurements were performed according to current recommendations of the European Association of Cardiovascular Imaging and the American Society of Echocardiography guidelines [12].

### 2.3. Clinical Endpoints and Follow-Up

All patients were followed up for the endpoint of all-cause mortality, obtained from hospital records linked to the governmental death registry. When patients underwent aortic valve replacement (AVR), it was also systematically noted.

### 2.4. Statistical Analysis

Continuous data are expressed as mean ± standard deviation if normally distributed and median and inter-quartile range (IQR) if non-normally distributed. Data were evaluated for normality using the Shapiro–Wilk test, and the homogeneity of variances was evaluated with Levene’s test. Categorical variables are expressed as frequencies and percentages.

Receiver operating characteristic (ROC) curve analysis was performed to identify the optimal Zva threshold associated with all-cause mortality. The optimal cutoffs, derived from the maximal Youden index, were used to stratify patients into two groups.

The unpaired 2-tailed *t*-test was used to compare normally distributed continuous variables between the two groups. When non-normally distributed, the Mann–Whitney U-test was used to compare continuous variables, and the chi-square test was used for the comparison of categorical variables. Differences in symptoms and LV remodeling were analyzed according to the Zva threshold.

Univariable Cox regression analysis was performed to identify the factors associated with all-cause mortality, presenting hazard ratios (HRs) with 95% confidence interval (CI). Proportional hazards assumptions were tested using Schoenfeld residuals. The association between Zva thresholds and all-cause death was also assessed using Kaplan–Meier survival analysis adjusted for relevant clinical and echocardiographic covariates. Separate multivariable analyses were performed to adjust for clinical and echocardiographic parameters, given the limited number of events.

All hypothesis tests were two-sided with a significance threshold of 0.05. In the Kaplan–Meier survival analysis, variables deemed clinically relevant were also adjusted for. All statistical analyses were performed using SPSS statistical software, version 29.0 (IBM, Armonk, NY, USA). This study was designed and reported in accordance with the Strengthening the Reporting of Observational Studies in Epidemiology (STROBE) guidelines.

## 3. Results

### 3.1. Clinical Characteristics of the Study Population

A total of 147 patients with BAV and severe AS were included. Table 1 presents the baseline demographic and clinical characteristics of the overall study population. Overall, the mean age was 63 ± 13 years, and 59% of patients were male. Hypertension was present in 58 patients (41%), while 42 patients (31%) had a history of smoking, and coronary artery disease (CAD) was present in 20 patients (19%). Overall, 28 patients (20%) were in NYHA functional class ≥ III.

During a median follow-up of 9.8 years (IQR, 8.0–12.9 years), 24 patients (16%) died, and 117 patients (80%) underwent AVR. The optimal cut-off value of Zva associated with all-cause mortality, determined by the maximal Youden index, was 5 mmHg/mL/m^2^ (AUC 0.64).

**Figure 1:**
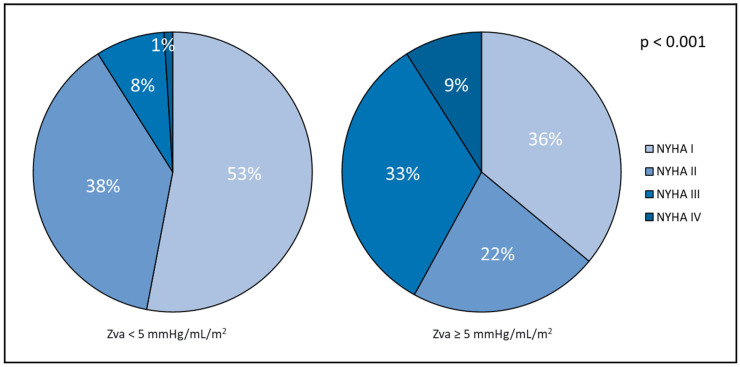
NYHA functional class based on Zva threshold. For abbreviations, refer to Table 1.

As shown in Table 1, patients with a Zva ≥ 5 mmHg/mL/m^2^ (*n* = 48, 30%) were significantly older (69 ± 13 years vs. 61 ± 13 years; *p* < 0.001), had a higher prevalence of arterial hypertension (54% vs. 34%; *p* = 0.023), diabetes mellitus (23% vs. 6%; *p* = 0.003), and CAD (30% vs. 13%; *p* = 0.028), as well as more advanced symptoms (NYHA class III-IV 41% vs. 9%; *p* < 0.001) and a lower estimated glomerular filtration rate (eGFR) (Figure 1).

### 3.2. Echocardiographic Characteristics of the Study Population

Echocardiographic characteristics of the overall study population, stratified by Zva threshold, are presented in Table 2. In the overall cohort, the median LVEF was 65% (IQR 55–70), and 88 patients (77%) had an LVEF ≥ 55%. Mean LV GLS was 16.0 ± 2.8%, and the median left atrial volume index (LAVI) was 33 (IQR 27–41) mL/m^2^.

**Figure 2:**
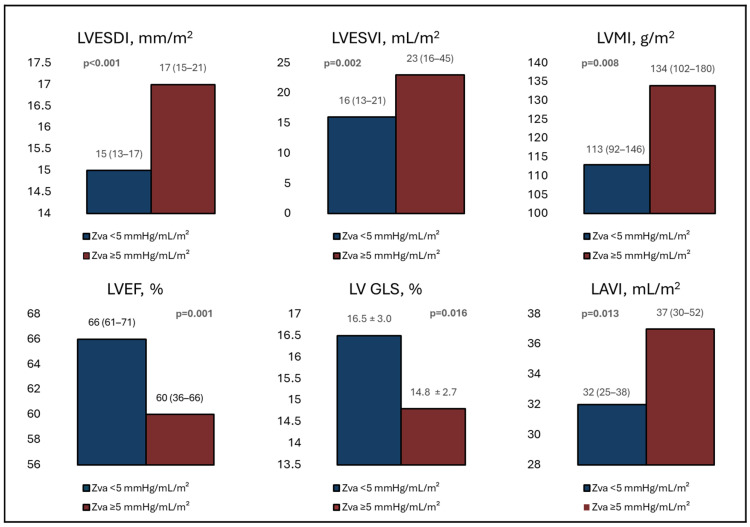
Echocardiographic parameters of cardiac remodeling and function according to Zva threshold. For abbreviations, refer to Table 2.

Patients with a Zva ≥ 5 mmHg/mL/m^2^ presented with worse LV systolic function, including a lower median LVEF (60% (IQR 36–66) vs. 66% (IQR 61–71); *p* = 0.001). These patients also had a significantly lower LV SVi (29 mL/m^2^ (IQR 26–34) vs. 45 mL/m^2^ (IQR 41–50); *p* < 0.001), more impaired LV GLS (14.8 ± 2.7% vs. 16.5 ± 3.0%; *p* = 0.016) and larger LAVI (37 mL/m^2^ (30–52) vs. 32 mL/m^2^ (IQR 25–38); *p* = 0.013) (Figure 2).

Patients with a Zva ≥ 5 mmHg/mL/m^2^ showed significantly larger LV end-systolic dimensions compared to those with a Zva < 5 mmHg/mL/m^2^. These differences remained significant after indexing for BSA (LVESDi 1.7 cm/m^2^ (IQR 1.5–2.1) vs. 1.5 cm/m^2^ (IQR 1.3–1.7); *p* < 0.001, LVESVi 23 mL/m^2^ (IQR 16–45) vs. 16 mL/m^2^ (IQR 13–21); *p* = 0.002). Furthermore, these patients had a higher LVMi (134 g/m^2^ (IQR 102–180) vs. 113 g/m^2^ (IQR 92–146); *p* = 0.008) and a greater prevalence of LV hypertrophy (74% vs. 49%; *p* = 0.007).

### 3.3. Clinical Outcomes and Association with Zva

During follow-up, all-cause mortality was significantly higher among patients with Zva ≥ 5 mmHg/mL/m^2^ as compared to those with lower Zva (29% vs. 10%; *p* = 0.003). However, the number of patients who underwent AVR during the follow-up did not differ between the groups (79% vs. 80%; *p* = 0.929).

In univariate Cox regression analysis, Zva ≥ 5 mmHg/mL/m^2^ was associated with a 3.6-fold increased risk of all-cause mortality (HR 3.609; 95% CI 1.541–8.545; *p* = 0.003) (Table S1). Other variables significantly associated with all-cause death included age, arterial hypertension, diabetes mellitus, CAD, LVESD, LVESV, as well as indexed LVESD, LVESV, LVEDV, and LVM.

**Figure 3:**
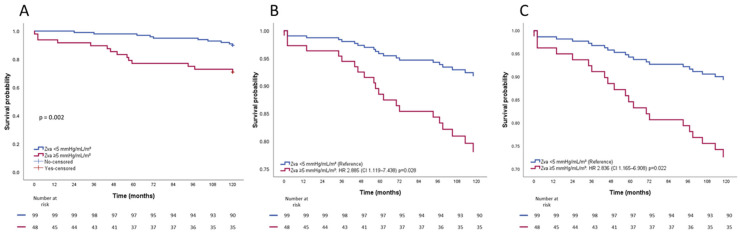
Kaplan–Meier curves for overall survival according to Zva threshold. (**A**). Unadjusted Kaplan–Meier curve. (**B**). Adjusted for age and NYHA functional class. (**C**). Adjusted for LVMi and LVESDi. For abbreviations, refer to Table 1 and Table 2.

Kaplan–Meier survival analysis showed a significantly worse 10-year survival in patients with Zva ≥ 5 mmHg/mL/m^2^ as compared to those below this threshold (73% vs. 91%, log-rank *p* = 0.002) (Figure 3A). After adjusting for clinical covariates (age and NYHA class), patients with Zva ≥ 5 mmHg/mL/m^2^ had a worse 10-year survival (HR 2.885; 95% CI 1.119–7.438; *p* = 0.028) (Figure 3B). This association persisted after additional adjustment for AVA (HR 3.081; 95% CI 1.158–8.193; *p* = 0.024) and also for echocardiographic measures of LV remodeling, including LVMi and LVESDi (HR 2.836; 95% CI 1.165–6.908; *p* = 0.022) (Figure 3C).

### 3.4. Interaction Analysis in Asymptomatic Patients

Among all 68 asymptomatic (NYHA I) patients (46%) in this study population, Zva ≥ 5 mmHg/mL/m^2^ was associated with a significantly increased risk of 10-year all-cause mortality (HR 6.306; CI 1.503–26.454; *p* = 0.012). However, interaction analysis showed no significant difference between Zva ≥ 5 mmHg/mL/m^2^ and mortality among symptomatic and asymptomatic patients (NYHA I vs. NYHA II-IV: interaction *p* = 0.780).

## 4. Discussion

The main findings of this cohort study of BAV patients with severe AS can be summarized as follows: (1) The optimal Zva threshold associated with all-cause mortality was 5 mmHg/mL/m^2^; (2) Zva ≥ 5 mmHg/mL/m^2^ was associated with more advanced symptoms; (3) more adverse LV remodeling and worse LV systolic function; and (4) after adjustment for relevant clinical and echocardiographic covariates, Zva ≥ 5 mmHg/mL/m^2^ remained independently associated with worse long-term survival.

### 4.1. Zva and Symptoms

Previous studies have shown that an increased Zva predicts symptom onset, unfavorable long-term quality of life, and reduced exercise capacity [3,14]. In a retrospective study by Lancellotti et al., including 163 patients with significant (≥moderate) AS, Zva independently predicted progression towards symptoms, suggesting that even among asymptomatic patients, increased Zva may identify patients at a higher risk who might benefit from earlier intervention [14]. Consistently, the present study extends these observations to patients with BAV and severe AS, in whom Zva ≥5 mmHg/mL/m^2^ was associated with more advanced symptoms.

Although Zva ≥ 5 mmHg/mL/m^2^ was associated with increased all-cause mortality in a subgroup of asymptomatic patients, no significant interaction between Zva ≥ 5 mmHg/mL/m^2^ and symptomatic status was found. Therefore, symptoms alone may not fully capture the risk of mortality in this specific patient population, extending previous hypotheses to a BAV-specific cohort and suggesting that even in the absence of symptoms, BAV patients with severe AS and increased Zva may benefit from earlier valve intervention.

### 4.2. Zva and LV Remodeling

Zva integrates valvular load (AV MPG) and arterial load (SBP) indexed to LV SVi, thereby approximating the global LV hemodynamic burden in severe AS. In patients with TAV-related severe AS, increased Zva has been associated with increased LV wall stress, promoting LV concentric remodeling and LV hypertrophy, with subsequent progression to LV systolic dysfunction [15]. Hachicha et al., in an asymptomatic cohort of 544 patients with at least moderate AS, showed significant differences in LV function and remodeling according to Zva level, with higher Zva associated with lower LVEF, larger end-diastolic linear and volumetric dimensions, and greater LVM and RWT [4]. Furthermore, LV hypertrophy and increased LVMi were independent predictors of all-cause mortality. In a smaller cohort of 82 patients with severe AS and preserved LVEF, Marechaux et al. further demonstrated an inverse correlation between increased Zva and LV GLS, and a positive correlation with LVMi and LVEDVi [5]. Mechanistically, increased Zva imposes a persistently increased global LV afterload, which may accelerate LV dilation and increase wall stress. This progressive impairment of LV contractile reserve likely contributes to adverse LV remodeling, worse LV systolic function, more advanced heart failure-related symptoms, and ultimately worse long-term survival.

In patients with BAV, in whom the relative contributions of valvular and arterial load may be heterogeneous, Zva may unmask a disproportionate afterload not fully captured by AVA or transvalvular gradients alone [5,16]. Consistent with the findings in TAV, patients with Zva ≥ 5 mmHg/mL/m^2^ in the present cohort exhibited more advanced LV remodeling and worse LV systolic function. These patients had larger LV linear and volumetric dimensions, and higher LVM, even after indexing for BSA, along with larger LAVI, lower LVEF, and worse LV GLS. Notably, indexed and non-indexed aortic diameters (SOV and AA) were similar across the Zva groups, indicating that the adverse prognostic implications of increased Zva are independent of aortopathy.

### 4.3. Zva and Clinical Outcomes

Prior studies, predominantly in TAV cohorts, have linked increased Zva to adverse outcomes, with proposed thresholds ranging from 4.5 to 5 mmHg/mL/m^2^ [3,4,14,17,18,19]. Lancelotti et al., for instance, reported a Zva threshold of ≥ 4.9 mmHg/mL/m^2^ as a strong predictor of adverse events [14]. Furthermore, Zva levels between 3.5–4.5 mmHg/mL/m^2^ have previously been proposed as thresholds for intermediate Zva by Hachicha et al. in a TAV cohort [4]. The present study, to our knowledge, is the first to confirm and extend these observations to a BAV-specific cohort, identifying 5 mmHg/mL/m^2^ as the optimal threshold above which long-term mortality risk is significantly increased.

In a cohort of 676 patients with preserved LVEF and severe AS, Magne et al. reported AVR rates comparable to those observed in the present study (91% vs. 80%, respectively), with no significant differences according to Zva strata [18]. Similarly, in our cohort, AVR rates did not differ between patients with Zva ≥ 5 mmHg/mL/m^2^ and those with Zva < 5 mmHg/mL/m^2^. Despite comparable rates of AVR, patients with increased Zva experienced significantly worse long-term survival. These findings suggest that Zva may help identify a subgroup of patients with a disproportionately increased global LV afterload who remain at higher risk despite similar rates of AVR and who may therefore benefit from closer monitoring, earlier consideration of AVR, and optimization of modifiable contributors to arterial load, including blood pressure control.

### 4.4. Limitations

The present study has limitations due to its retrospective observational design. SBP was not always measured at the exact time of echocardiographic assessment (although always at the corresponding outpatient visit), which may have influenced the calculation of Zva in a small proportion of patients. Other variables, namely comorbidities such as diabetes and kidney disease, may have influenced the results. However, the limited number of outcome events in the BAV cohort did not allow for building a unique adjusted survival analysis, necessitating the use of multiple adjusted models. Finally, all-cause mortality was selected as the most robust primary endpoint, as the specific cause of death was not systematically reported.

Prospective, multi-center studies with standardized, near-simultaneous acquisition of SBP, AV MPG, and SVi are therefore warranted to improve the accuracy of Zva and to validate the 5 mmHg/mL/m^2^ threshold as a predictor of long-term outcomes in patients with BAV and AS.

## 5. Conclusions

Among patients with BAV and severe AS, a Zva threshold of 5 mmHg/mL/m^2^ was independently associated with more advanced symptoms, adverse LV remodeling, and worse long-term survival. These findings suggest that Zva may serve as an additional tool for risk stratification in this population. Serial assessment of Zva may help guide the timing of intervention, particularly in asymptomatic patients and those with preserved LVEF.

## Figures and Tables

**Table 1:** Baseline clinical characteristics of the overall cohort and stratified by Zva threshold.

Variable	Total Population *n* = 147	Zva < 5 mmHg/mL/m^2^ *n* = 99	Zva ≥ 5 mmHg/mL/m^2^ *n* = 48	*p*-Value
**Age, mean ± SD, years**	63 ± 13	61 ± 13	69 ± 13	**<0.001**
**Male gender (%)**	86 (59)	61 (62)	25 (52)	0.271
**BSA, mean ± SD, m^2^**	1.93 ± 0.21	1.92 ± 0.19	1.95 ± 0.24	0.357
**Smoking (%)**	42 (31)	34 (37)	8 (18)	**0.024**
**Hypertension (%)**	58 (41)	33 (34)	25 (54)	**0.023**
**Dyslipidemia (%)**	51 (35)	36 (36)	15 (32)	0.598
**Diabetes mellitus (%)**	15 (11)	5 (6)	10 (23)	**0.003**
**Coronary artery disease (%)**	20 (19)	8 (13)	12 (30)	**0.028**
**COPD (%)**	8 (8)	4 (6)	4 (10)	0.476
**Family history of CAD (%)**	21 (21)	11 (18)	10 (26)	0.464
**NYHA III or IV (%)**	28 (20)	9 (9)	19 (41)	**<0.001**
**eGFR** **, median (IQR), mL/min/1.73m^2^**	81 (67–92)	84 (71–97)	71 (55–82)	**<** **0.001**

Values are presented as mean ± SD or *n* (%). BSA: body surface area; CAD: coronary artery disease; COPD: chronic obstructive pulmonary disease; eGFR: estimated glomerular filtration rate; NYHA: New York Heart Association; SD: standard deviation; Zva: valvulo-arterial impedance.

**Table 2:** Baseline echocardiographic characteristics of the overall cohort and stratified by Zva threshold.

Variable	Total Population *n* = 147	Zva < 5 mmHg/mL/m^2^ *n* = 99	Zva ≥ 5 mmHg/mL/m^2^ *n* = 48	*p*-Value
***Left ventricular and atrial parameters***				
**LV ESD, median (IQR), cm**	3.0 (2.5–3.5)	2.8 (2.5–3.2)	3.3 (2.8–4.3)	**0.001**
**LV ESD indexed, median (IQR), cm/m^2^**	1.5 (1.4–1.8)	1.5 (1.3–1.7)	1.7 (1.5–2.1)	**<0.001**
**LV ESV, median (IQR), mL**	35 (23–53)	33 (22–43)	46 (30–85)	**0.003**
**LV ESV indexed, median (IQR), mL/m^2^**	18 (13–26)	16 (13–21)	23 (16–45)	**0.002**
**IVST, mean ± SD, mmHg, cm**	1.3 ± 0.3	1.3 ± 0.3	1.4 ± 0.3	**0.026**
**PWT, mean ± SD, cm**	1.2 ± 0.3	1.2 ± 0.2	1.3 ± 0.3	**0.049**
**LVEF, median (IQR), %**	65 (55–70)	66 (61–71)	60 (36–66)	**0.001**
**LVEF ≥ 55% (%)**	88 (77)	67 (89)	21 (54)	**<0.001**
**LV SVi, median (IQR), mL/m^2^**	41 (33–47)	45 (41–50)	29 (26–34)	**<0.001**
**LV GLS, mean ± SD, %**	16.0 ± 2.8	16.5 ± 3.0	14.8 ± 2.7	**0.016**
**LAVI, median (IQR), mL/m^2^**	33 (27–41)	32 (25–38)	37 (30–52)	**0.013**
**LAVI ≥ 34 mL/m^2^ (%)**	52 (48)	27 (39)	25 (64)	**0.011**
**Mitral inflow E/A ratio, median (IQR)**	0.9 (0.7–1.4)	0.9 (0.7–1.4)	0.9 (0.7–1.2)	0.569
***LV remodeling parameters and patterns***				
**LV mass, median (IQR), g**	231 (176–298)	213 (167–276)	253 (201–316)	**0.007**
**LV mass indexed, median (IQR), g/m^2^**	116 (94–149)	113 (92–146)	134 (102–180)	**0.008**
**RWT, mean ± SD**	0.52 ± 0.14	0.51 ± 0.13	0.53 ± 0.17	0.375
**LV hypertrophy (%)**	74 (57)	43 (49)	31 (74)	**0.007**
**LV remodeling pattern (%)**				0.061
**Normal geometry**	19 (15)	15 (17)	4 (10)	
**Concentric remodeling**	37 (29)	30 (34)	7 (17)	
**Concentric hypertrophy**	61 (47)	36 (41)	25 (60)	
**Eccentric hypertrophy**	13 (10)	7 (8)	6 (14)	
***Aortic valve and aorta parameters***				
**AV Vmax, mean ± SD, m/s**	4.2 ± 0.9	4.4 ± 0.8	4.0 ± 1.0	0.051
**AVA, median (IQR), cm^2^**	0.8 (0.7–1.0)	0.9 (0.8–1.0)	0.7 (0.6–0.9)	**<0.001**
**AVA indexed, median (IQR) cm^2^/m^2^**	0.43 (0.36–0.49)	0.44 (0.40–0.50)	0.37 (0.30–0.47)	**<0.001**
**SOV diameter, mean ± SD, mm**	34 ± 5	34 ± 5	33 ± 5	0.355
**SOV diameter indexed, mean ± SD, mm/m^2^**	18 ± 3	18 ± 3	17 ± 3	0.168
**AA diameter, mean ± SD, mm**	39 ± 6	39 ± 5	38 ± 6	0.794
**AA diameter indexed, mean ± SD, mm/m^2^**	20 ± 3	20 ± 3	20 ± 4	0.498

Values are presented as mean ± SD, median (IQR), or *n* (%). AA: ascending aorta; AV: aortic valve; AVA: aortic valve area; ESD: end-systolic diameter; ESV: end-systolic volume; GLS: global longitudinal strain; IQR: interquartile range; IVST: interventricular septum thickness; LAVI: left atrial volume index; LV: left ventricular; LVEF: left ventricular ejection fraction; PWT: posterior wall thickness; RWT: relative wall thickness; SD: standard deviation; SOV: sinus of Valsalva; SVi: stroke volume index; Vmax: peak velocity; Zva: valvulo-arterial impedance.

## Data Availability

The data that support the findings of this study are not openly available due to reasons of sensitivity and are available from the corresponding author upon reasonable request.

## Supplementary Materials

The following supporting information can be downloaded at: https://www.mdpi.com/article/10.3390/jcdd13040163/s1, Table S1: Univariate Cox proportional hazard analysis for all-cause mortality in the entire cohort.
