# Supplementary material for: Valvulo-Arterial Impedance in Patients with Severe Aortic Stenosis and Bicuspid Aortic Valve
Source: J Cardiovasc Dev Dis. 2026 Apr 9;13(4):163. doi: 10.3390/jcdd13040163 (PMC13115660; doi:10.3390/jcdd13040163)
Supplement: Supplementary file 1 [file jcdd-13-00163-s001.zip › Table S1.pdf]

Table S1. Univariate Cox proportional hazard analysis for all-cause mortality in the entire cohort.

Variable	HR (95% CI)	p-value
Age, years	1.080 (1.040-1.122)	**<0.001**
Male gender	0.870 (0.376-2.013)	0.745
Hypertension	2.568 (1.063-6.200)	**0.036**
Diabetes mellitus	2.801 (1.033-7.596)	**0.043**
Coronary artery disease	3.633 (1.381-9.560)	**0.009**
eGFR, mL/min/1.73m^2	0.970 (0.951-0.989)	**0.002**
NYHA ≥ III	2.416 (0.973-5.997)	0.057
LVEF ≥ 55%	0.461 (0.184-1.157)	0.099
LV EDD, cm	1.414 (0.883-2.263)	0.149
LV ESD, cm	1.415 (1.001-2.002)	**0.050**
LV EDV, mL	1.008 (1.000-1.017)	0.054
LV ESV, mL	1.011 (1.002-1.019)	**0.012**
LV EDD indexed, cm/m^2	1.955 (0.862-4.434)	0.108
LV ESD indexed, cm/m^2	1.969 (1.076-3.603)	**0.028**
LV EDV indexed, mL/m^2	1.022 (1.003-1.040)	**0.023**
LV ESV indexed, mL/m^2	1.025 (1.008-1.043)	**0.004**
LV GLS, %	0.936 (0.773-1.135)	0.502
LV mass, g	1.005 (1.001-1.009)	**0.010**
LV mass indexed, g/m^2	1.009 (1.002-1.017)	**0.008**
SOV indexed, mm/m^2	1.105 (0.952-1.283)	0.189
Zva, mmHg/mL/m^2	1.350 (1.106-1.648)	**0.003**
Zva ≥5 mmHg/mL/m^2	3.609 (1.541-8.545)	**0.003**
AVR*	0.880 (0.325-2.385)	0.801

AVR*: aortic valve replacement as a time-dependent covariate, CI: confidence interval, EDD: end-diastolic diameter, EDV: end-diastolic volume, eGFR: estimated glomerular filtration rate, ESD: end-systolic diameter, ESV: end-systolic volume, GLS: global longitudinal strain, HR: hazard ratio, LV: left ventricular, LVEF: left ventricular ejection fraction, NYHA: New York Heart Association, SOV: sinus of Valsalva, Zva: valvulo-arterial impedance.
